# Microbial Prevalence, Diversity and Abundance in Amniotic Fluid During Preterm Labor: A Molecular and Culture-Based Investigation

**DOI:** 10.1371/journal.pone.0003056

**Published:** 2008-08-26

**Authors:** Daniel B. DiGiulio, Roberto Romero, Harold P. Amogan, Juan Pedro Kusanovic, Elisabeth M. Bik, Francesca Gotsch, Chong Jai Kim, Offer Erez, Sam Edwin, David A. Relman

**Affiliations:** 1 Department of Medicine, Stanford University School of Medicine, Stanford, California, United States of America; 2 Veterans Affairs Palo Alto Health Care System, Palo Alto, California, United States of America; 3 Perinatology Research Branch, National Institute of Child Health and Human Development (NICHD), National Institutes of Health (NIH), Bethesda, Maryland, and Detroit, Michigan, United States of America; 4 Center for Molecular Medicine and Genetics, Wayne State University School of Medicine, Detroit, Michigan, United States of America; 5 Department of Obstetrics and Gynecology, Wayne State University School of Medicine, Detroit, Michigan, United States of America; 6 Department of Microbiology and Immunology, Stanford University, Stanford, California, United States of America; 7 Department of Pathology, Wayne State University School of Medicine, Detroit, Michigan, United States of America; University of Queensland Centre for Clinical Research, Australia

## Abstract

**Background:**

Preterm delivery causes substantial neonatal mortality and morbidity. Unrecognized intra-amniotic infections caused by cultivation-resistant microbes may play a role. Molecular methods can detect, characterize and quantify microbes independently of traditional culture techniques. However, molecular studies that define the diversity and abundance of microbes invading the amniotic cavity, and evaluate their clinical significance within a causal framework, are lacking.

**Methods and Findings:**

In parallel with culture, we used broad-range end-point and real-time PCR assays to amplify, identify and quantify ribosomal DNA (rDNA) of bacteria, fungi and archaea from amniotic fluid of 166 women in preterm labor with intact membranes. We sequenced up to 24 rRNA clones per positive specimen and assigned taxonomic designations to approximately the species level. Microbial prevalence, diversity and abundance were correlated with host inflammation and with gestational and neonatal outcomes. Study subjects who delivered at term served as controls. The combined use of molecular and culture methods revealed a greater prevalence (15% of subjects) and diversity (18 taxa) of microbes in amniotic fluid than did culture alone (9.6% of subjects; 11 taxa). The taxa detected only by PCR included a related group of fastidious bacteria, comprised of *Sneathia sanguinegens*, *Leptotrichia amnionii* and an unassigned, uncultivated, and previously-uncharacterized bacterium; one or more members of this group were detected in 25% of positive specimens. A positive PCR was associated with histologic chorioamnionitis (adjusted odds ratio [OR] 20; 95% CI, 2.4 to 172), and funisitis (adjusted OR 18; 95% CI, 3.1 to 99). The positive predictive value of PCR for preterm delivery was 100 percent. A temporal association between a positive PCR and delivery was supported by a shortened amniocentesis-to-delivery interval (adjusted hazard ratio 4.6; 95% CI, 2.2 to 9.5). A dose-response association was demonstrated between bacterial rDNA abundance and gestational age at delivery (r^2^ = 0.42; P<0.002).

**Conclusions:**

The amniotic cavity of women in preterm labor harbors DNA from a greater diversity of microbes than previously suspected, including as-yet uncultivated, previously-uncharacterized taxa. The strength, temporality and gradient with which these microbial sequence types are associated with preterm delivery support a causal relationship.

## Introduction

Preterm birth is the leading cause of neonatal mortality worldwide [1], yet its underlying etiologies remain largely unknown [2], [3]. Mortality exhibits an inverse relationship with gestational age, such that early preterm neonates (e.g., less than 32 gestational weeks) account for the vast majority of deaths [4]–[6]. Consequently, the need for insights into factors contributing to early preterm delivery is particularly acute.

A strong body of evidence suggests that occult intra-uterine infection plays a major role in preterm labor and delivery [7]. These infections are thought to escape detection primarily because they are subclinical, but also because they may be caused by cultivation-resistant microbes [8]. Fastidious bacterial taxa, such as mycoplasmas, are among those most commonly implicated in preterm birth [8]–[11], but the specialized techniques required to cultivate them reliably are seldom used in clinical settings [9]–[11]. Other microbial groups, including the majority of species in many ecosystems, cannot be cultivated with current methods [12].

Molecular methods such as polymerase chain reaction (PCR) can detect microbes independently of culture. Moreover, broad-range PCR assays that amplify highly-conserved but phylogenetically-informative gene sequences can identify microbes across broad taxonomic levels, including previously uncharacterized species. By overcoming investigator biases inherent to more specific detection methods, sequencing of broad-range PCR products has emerged as a powerful approach for revealing previously unsuspected microbial diversity in various anatomic niches in human health [13] and disease [14], and for characterizing uncultivated human pathogens [15].

The application of broad-range PCR to amniotic fluid has been limited to date. In particular, molecular investigations that characterize the microbial diversity of the amniotic cavity in a systematic manner, and evaluate findings within a coherent causal framework, are lacking. As an early step in defining the potential role of diverse microbial sequence types, including uncultivated taxa, in preterm delivery, we conducted a broad-range molecular investigation. In parallel with traditional amniotic fluid cultures, we used qualitative and quantitative PCR assays to amplify, identify and quantify ribosomal DNA (rDNA) of bacteria, fungi and archaea from amniotic fluid of patients with spontaneous preterm labor and intact membranes. We examined sequence diversity in samples with detectable rDNA, and correlated findings with pre-specified measures of host inflammation, as well as pregnancy and neonatal outcome. We sought evidence for the types of associations that have been proposed as alternatives to Koch's postulates for inferring causality from molecular data (e.g., associations of space, time and dose) [16]. Here, we report the prevalence, diversity and abundance of microbes in amniotic fluid during preterm labor, and their clinical significance.

## Methods

### Study population

A retrospective cohort study was conducted by searching our clinical database to identify patients with the diagnosis of spontaneous preterm labor with intact membranes, enrolled at Hutzel Women's Hospital (Detroit, MI) between October 1998 and December 2002, who provided written informed consent and for whom an adequate volume of amniotic fluid was available with which to conduct these research assays after clinically-indicated conventional analyses were completed. Patients were included if they met the following criteria: 1) singleton gestation; 2) gestational age between 18 and 35 weeks; and 3) had an amniocentesis with microbiological studies of amniotic fluid. Patients were excluded from the study if: 1) rupture of the chorioamniotic membranes occurred before amniotic fluid collection; 2) the amniotic fluid was collected transvaginally; 3) delivery occurred elsewhere and/or clinical data were unavailable; and 4) a major fetal congenital anomaly was present.

A total of 166 subjects were included in this study. The pre-specified control group consisted of study subjects who delivered at term. All women provided written informed consent prior to the collection of biological samples. The utilization of samples for research purposes was approved by the Institutional Review Boards of Wayne State University, the National Institute of Child Health and Human Development (NICHD/NIH/DHHS), and Stanford University.

### Definitions

Preterm labor was diagnosed by the presence of at least two regular uterine contractions every 10 minutes associated with cervical changes that required hospital admission before 37 weeks of gestation. Preterm delivery was defined as delivery before 37 weeks of gestation. Clinical chorioamnionitis was diagnosed according to criteria previously proposed [17], including maternal temperature of ≥37.8°C and two or more of the following criteria: uterine tenderness, malodorous vaginal discharge, maternal leukocytosis (≥15000 cells/mm^3^), maternal tachycardia (>100 beats/min) and fetal tachycardia (>160 beats/min). Histologic chorioamnionitis was diagnosed based on the presence of inflammatory cells in the chorionic plate and/or chorioamniotic membranes. Acute funisitis was diagnosed by the presence of neutrophils in the wall of the umbilical vessels and/or Wharton's jelly using criteria previously described [18].

### Sampling procedures

Enrolled subjects underwent transabdominal, ultrasound-guided amniocentesis, which is within the standard of care at Hutzel Women's Hospital for evaluating possible microbial invasion of the amniotic cavity of patients with spontaneous preterm labor. Amniotic fluid was immediately transported in a capped sterile syringe to the clinical laboratory where it was cultured for aerobic and anaerobic bacteria, including genital mycoplasmas. White blood cell (WBC) count and Gram stain of amniotic fluid were also performed shortly after collection. Amniotic fluid not required for clinical assessment was centrifuged for 10 minutes at 4°C shortly after the amniocentesis, and the supernatant was aliquoted and stored at −70°C until analysis. Amniotic fluid IL-6 concentrations were determined after delivery for research purposes, and these results were not used in patient management. A flowchart of our overall approach to amniotic fluid analysis is illustrated in Supporting Figure S1; detailed experimental methods, including microbiologic techniques and IL-6 quantitation, appear in Supporting Materials and Methods S1.

### Molecular analysis

Microbial genomic DNA was extracted from 200 µl of each amniotic fluid sample. Qualitative analysis was achieved by means of three separate broad-range end-point PCR assays targeting rDNA of either bacteria, fungi, or archaea. Cloned amplicons (up to 24 clones per positive PCR reaction) were bidirectionally sequenced, the sequences aligned, and then subjected to phylogenetic analysis (621 nucleotide positions). Quantitative rDNA analysis was achieved by means of three real-time PCR assays corresponding to each of the three microbial groups targeted by end-point PCR. Methodologic details of the key aspects of our molecular approach (including nucleic acid extraction, PCR assays, phylogenetic analysis, and contamination prevention) appear under Supporting Materials and Methods S1; PCR primer and probe sequences appear in Supporting Table S4.

### Outcome Measures

In order to assess the clinical significance of the microbes detected with molecular methods, pre-specified outcome variables from four broad categories were measured: 1) markers of intra-amniotic inflammation (including amniotic fluid WBC count [19] and IL-6 concentration [20]); 2) histopathologic evidence of maternal or fetal inflammation (including chorioamnionitis and funisitis); 3) pregnancy outcomes (including gestational age at delivery, and amniocentesis-to-delivery interval); and 4) neonatal outcome. Neonatal outcome was assessed by measuring composite neonatal morbidity and mortality, defined as the presence of one or more of the following: bronchopulmonary dysplasia, respiratory distress syndrome, necrotizing enterocolitis, intraventricular hemorrhage≥grade III, and sepsis, which were diagnosed according to previously described criteria [21], as well as respiratory failure requiring mechanical ventilation, and neonatal death.

### Statistical analysis

Statistical analyses were performed using ‘R’ (open source, www.r-project.org) version 2.4.1, including the ‘Epi’ and ‘Survival’ packages. For gestational outcomes, patients with an episode of spontaneous preterm labor who delivered at term served as controls. Positive predictive values were estimated by means of contingency table analysis. Differences in the median between groups were computed using the Mann Whitney *U* test when two groups were compared and the Kruskal-Wallis analysis of variance test when more than two groups were compared. Unadjusted and adjusted odds ratios (OR) for binary outcomes were estimated by simple and multiple logistic regression modeling, respectively. Correlation coefficients for continuous outcomes were estimated by means of least squares linear regression modeling. Time-to-event outcomes were modeled by Kaplan-Meier survival methods; differences between survival curves were evaluated by the Mantel-Haenszel log-rank test, and proportional hazard ratios were estimated by the method of Cox. For all analyses, a two-tailed P value <0.05 was considered significant. Additional details of the statistical methods used, including the calculation of false discovery rates for P values corresponding to multiple pairwise comparisons, appear under Supporting Materials and Methods S1.

## Results

**Figure 1:**
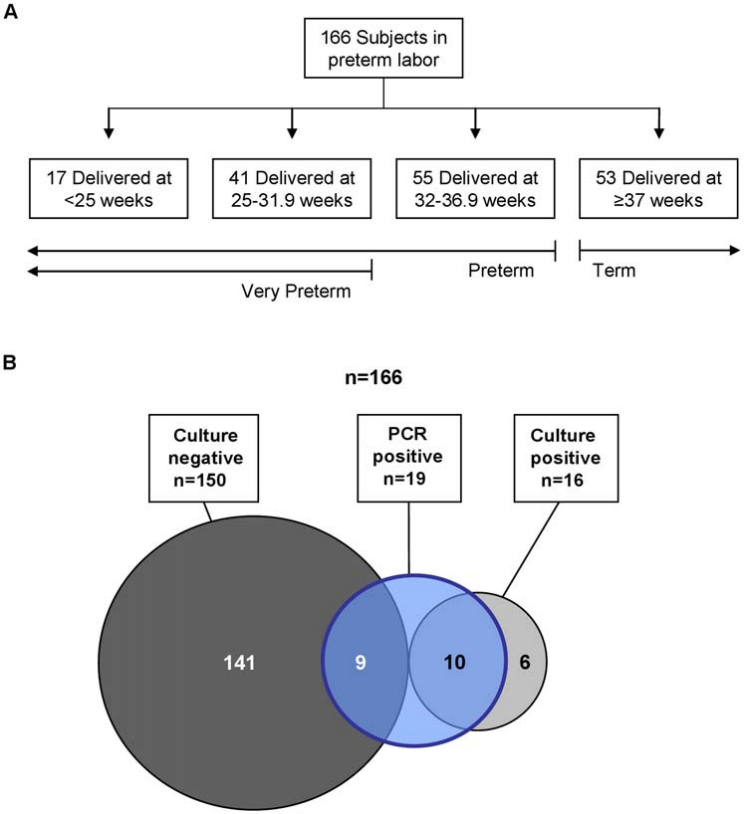
Gestational Outcomes and Amniotic Fluid Findings. Panel A shows outcomes of enrolled subjects. Panel B summarizes results of culture and PCR analysis of amniotic fluid. PCR refers to end-point PCR for bacteria, fungi and archaea. Culture refers to the use of routine cultivation methods for bacteria (aerobic, anaerobic and genital mycoplasmas) and fungi. Circle areas are not to scale.

Of 166 subjects, 53 (32%) delivered at term and 113 (68%) delivered preterm (Figure 1A). Ten patients (6%) had clinical chorioamnionitis. Baseline characteristics appear in Supporting Table S1.

### Microbial prevalence and diversity

Microbial invasion of the amniotic cavity – as defined by either a positive end-point PCR or culture of amniotic fluid – was found in 15% (25/166) of patients. Of these, bacteria were cultivated from 15 and a fungus from 1, whereas bacterial rDNA was amplified from 17 and fungal rDNA from 2. Six culture-positive samples were negative by PCR, and nine PCR-positive samples were negative by culture (Figure 1B). No archaea were detected. Viruses were not targeted by molecular methods; however, culture for cytomegalovirus (CMV) was performed on 79 samples. Of these, 2.5% (2/79) were positive; both subjects delivered full-term neonates.

**Figure 2:**
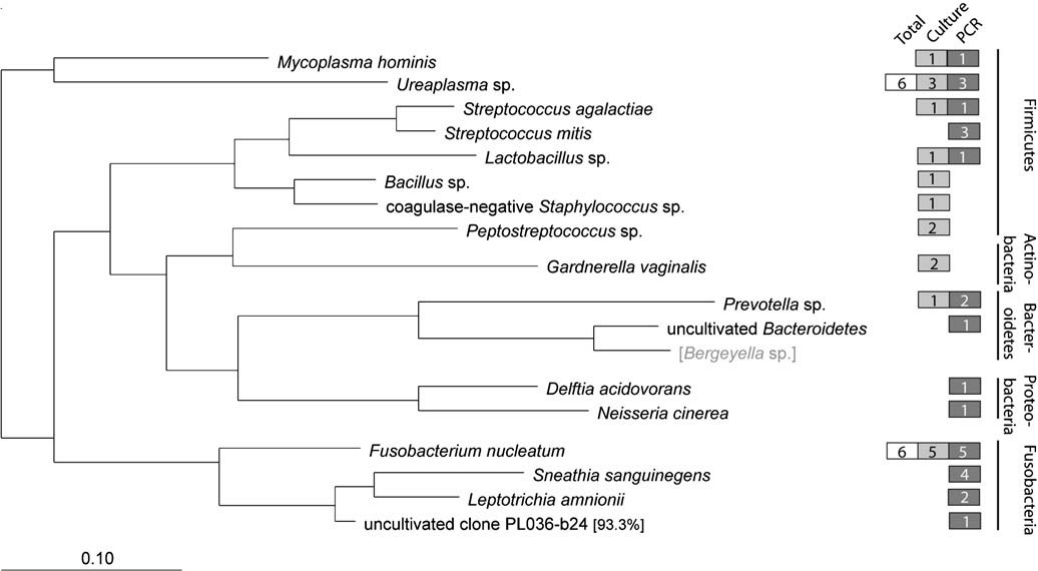
Microbial Diversity. Phylogeny of the 17 bacterial taxa identified in this study, based on a maximum likelihood algorithm. Colored boxes indicate the number of subjects who were positive for a given taxon by culture (gray) or PCR (blue) (some samples were polymicrobial). For most individual taxa, the larger of the two numbers in the corresponding gray or blue box represents the total number of positive subjects; for taxa where neither method detected all positive subjects, the total number is shown in the white box. A 99% sequence similarity cutoff threshold was used for phylotype assignment, which was based on 621 unambiguous nucleotide positions. *Bergeyella* sp. (bracketed and in gray type) is included as a reference species only and was not detected in the study population. A single fungal species, *Candida albicans*, was detected by culture in 1 subject and by PCR in 2 (data not shown). GenBank accession numbers of bacterial and fungal rDNA sequences from this study appear in Supporting Table S2.

Among the 25 patients whose amniotic fluid tested positive by culture or end-point PCR, 17 bacterial species (belonging to 5 phyla) and 1 fungal species were identified. Of the 17 bacterial taxa identified, 6 were detected by both culture and PCR (*Mycoplasma hominis*, *Ureaplasma* sp., *Streptococcus agalactiae*, *Lactobacillus* sp., *Prevotella* sp., and *Fusobacterium nucleatum*), 4 were detected by culture only (coagulase-negative *Staphylococcus* sp., *Bacillus* sp. not *anthracis*, *Peptostreptococcus* sp., and *Gardnerella vaginalis*), and 7 were detected by PCR only (*Streptococcus mitis*, uncultivated *Bacteroidetes* bacterium, *Delftia acidovorans*, *Neisseria cinerea*, *Sneathia* (formerly *Leptotrichia*) *sanguinegens*, *Leptotrichia amnionii*, and one phylotype (clone PL036-b24) with <95% rDNA sequence identity to its closest Genbank relative that appeared to represent a novel species) (Figure 2). The single fungal species identified was *Candida albicans*. The fastidious nature of many taxa detected only by PCR (e.g. *S. sanguinegens* and *L. amnionii*) supported the underlying premises of this study. Characteristics of individual subjects testing positive by PCR or culture, including GenBank accession numbers of rDNA sequences, are detailed in Supporting Table S2. We next investigated the relevance of molecular detection of these diverse taxa in the population as a whole by examining associations with host inflammation, and seeking evidence for categorical, temporal, and dose-response associations with preterm delivery.

### Association of intra-amniotic microbial DNA with host (maternal and fetal) inflammation

**Figure 3:**
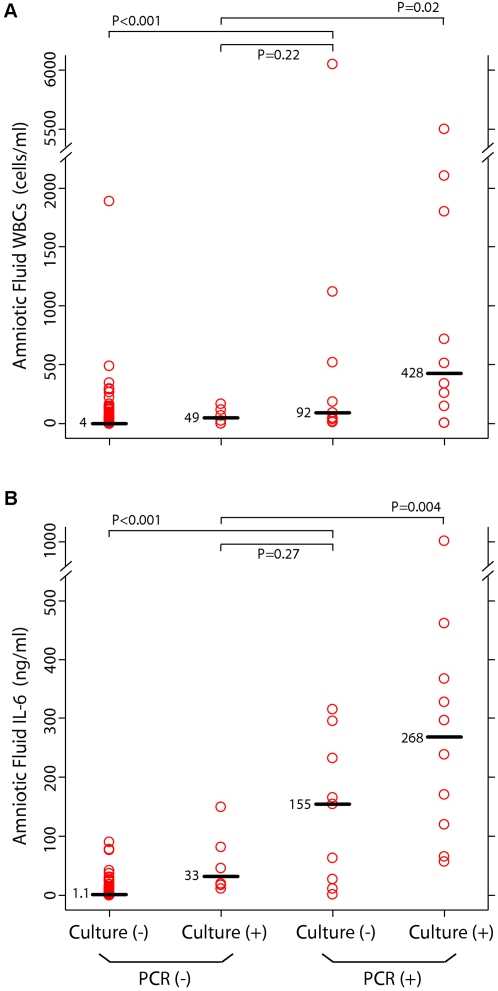
Correlation of PCR and Culture Results with Markers of Infection in Amniotic Fluid. Amniotic fluid concentrations of white blood cells (WBCs) (Panel A) and interleukin-6 (IL-6) (Panel B) as a function of PCR and culture results. Each circle represents one subject; each horizontal bar and adjacent number indicates the median value for that group. P values were calculated by means of the Mann-Whitney U test. PCR refers to end-point PCR for bacteria, fungi and archaea. Culture refers to the use of routine cultivation methods for bacteria (aerobic, anaerobic and genital mycoplasmas) and fungi.

A host inflammatory response has been implicated in the pathogenesis of preterm delivery [8]. In order to evaluate the association between microbial DNA and intra-amniotic inflammation, results from end-point PCR and culture were correlated with amniotic fluid WBC count and IL-6 concentration, which typically are elevated in the setting of infection [19], [21], [22]. Figure 3 shows that the median amniotic fluid WBC count and IL-6 concentration in the PCR-positive / culture-negative (P+/C−) group were higher than the median concentrations of each marker in the P−/C− group (P<0.001 for WBC and for IL-6), and equivalent to the median concentrations of each respective marker in the P−/C+ group (P = 0.22 for WBC; P = 0.27 for IL-6). These results indicate that the inflammatory response in the amniotic cavity of patients who are positive only by PCR is as robust as the inflammatory response in those who are positive only by culture. In addition, the median WBC count and IL-6 concentration in the P+/C+ group were higher than the median concentrations of each respective marker in the P−/C+ group (for WBC, P = 0.02, false discovery rate 152/1000; for IL-6, P = 0.004, false discovery rate 45/1000) (Figure 3), suggesting that a positive PCR result may be clinically significant even in the setting of a positive culture.

To evaluate associations of microbial DNA with inflammation of maternal and fetal tissues, results from end-point PCR and culture were correlated with histologic chorioamnionitis, which has been previously associated with preterm delivery [23], and with funisitis. Table 1 summarizes results from simple and multiple logistic regression modeling. After controlling for other covariates exhibiting statistically significant associations (see Table 1), the adjusted ORs of PCR and of culture for histological chorioamnionitis were 20 (95% CI, 2.4 to 172) and 8.6 (95% CI, 1.0 to 76), respectively; the adjusted ORs of PCR and of culture for funisitis were 18 (95% CI, 3.1 to 99) and 5.8 (95% CI, 1.1 to 32), respectively.

**Table 1:** Factors Associated with Histologic Chorioamnionitis, Funisitis, and Neonatal Morbidity and Mortality.

Variable	Histologic Chorioamnionitis* (n = 57)		Funisitis† (n = 48)		Composite Neonatal Morbidity and Mortality‡ (n = 63)	
	Univariate	Multivariate	Univariate	Multivariate	Univariate	Multivariate
Positive amniotic fluid PCR	37 (4.8–288)	20 (2.4–172)	24 (5.2–109)	18 (3.1–99)	16.2 (3.6–73)	5.2 (0.84–32)
Positive amniotic fluid culture	31 (4.0–243)	8.6 (1.0–76)	20 (4.2–91)	5.8 (1.1–32)	23 (2.9–179)	7.1 (0.66–76)
Maternal age	0.99 (0.93–1.0)	0.97 (0.90–1.0)	0.95 (0.89–1.0)	0.9 (0.84–0.98)	1.0 (0.97–1.1)	NA
Gestational age at amniocentesis	0.87 (0.80–0.94)	0.89 (0.81–0.97)	0.9 (0.81–0.96)	0.89 (0.83–0.98)	0.84 (0.78–92)	0.80 (0.72–0.88)
Cervical dilatation	1.1 (0.91–1.4)	NA	1.1 (0.90–1.4)	NA	1.4 (1.1–1.7)	1.6 (1.2–2.1)

Results are reported as odds ratios (95% confidence interval).

The table includes variables that exhibited a statistically significant association with one or more of the three measured outcomes. For each outcome, the multivariate model was comprised of those variables exhibiting a significant association in the univariate model. Other variables that were subject to univariate analysis but did not exhibit a significant association with any of these outcomes were: African-American race, nulliparity, previous preterm delivery, cigarette smoking, and recreational drug use.

* Diagnosed based on the presence of inflammatory cells in the chorionic plate and/or chorioamniotic membranes.

† Defined as the presence of neutrophils in the wall of the umbilical vessels and/or Wharton's jelly.

‡ Defined as the presence of any one or more of the following: bronchopulmonary dysplasia, respiratory distress syndrome, necrotizing enterocolitis, intraventricular hemorrhage of grade≥III, sepsis, respiratory failure requiring mechanical ventilation, and neonatal death.

NA Not applicable.

### Association of intra-amniotic microbial DNA with gestational and neonatal outcomes

**Figure 4:**
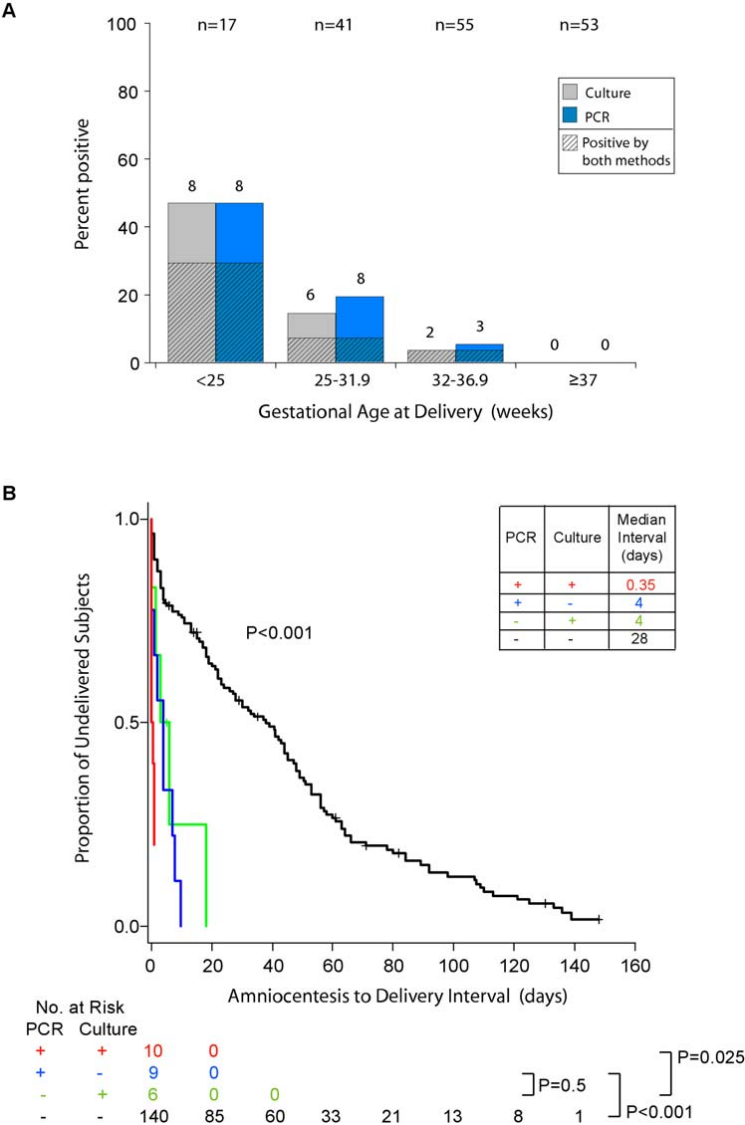
Gestational Outcomes According to Results of Culture and PCR. Panel A shows the proportion of subjects from each gestational age cohort who were positive by culture or PCR. The total of number of subjects in each cohort appears across the top of the graph, and the number of positive subjects appears above each bar. Panel B is a Kaplan-Meier analysis of amniocentesis-to-delivery interval according to culture and PCR results. Subjects in whom labor was augmented were censored and are represented by crosses. P values were calculated by means of the Mantel-Haenszel log-rank test. The inset table shows the median interval for each group. For both panels, PCR results refer to end-point PCR for bacteria, fungi and archaea, and culture refers to the use of routine cultivation methods for bacteria (aerobic, anaerobic and genital mycoplasmas) and fungi.

To evaluate the association of microbial DNA with gestational outcomes, results from end-point PCR and culture were correlated with gestational age at delivery. All subjects testing positive by either PCR or culture delivered preterm; this complete separation with respect to test result precluded logistic regression analysis. However, when the study population was stratified according to clinically-relevant gestational-age cut offs, the frequency distribution of positive PCR assays exhibited a clear inverse relationship with gestational age at delivery (Figure 4A). The positive predictive value of PCR for delivery before 37-, 32-, and 25-weeks was 100% for each time point (Table 2).

**Table 2:** Positive Predictive Value (PPV) of Amniotic Fluid PCR and Culture for Pregnancy Outcomes.

Outcome	Preterm delivery (<37 weeks)	Very preterm delivery (<32 weeks)	Extremely preterm delivery (<25 weeks)	Delivery within one day of amniocentesis
Prevalence	68% (113/166)	49% (58/118)	40% (17/43)	17% (28/166)
PPV of PCR	100% (19/19)	100% (16/16)	100% (8/8)	68% (13/19)
PPV of culture	100% (16/16)	100% (14/14)	89% (8/9)	69% (11/16)
PPV of PCR and culture combined	100% (10/10)	100% (8/8)	100% (5/5)	100% (10/10)

To evaluate the association of microbial DNA with neonatal outcomes, results from end-point PCR and culture were correlated with a composite of neonatal morbidity and mortality by means of simple and multiple logistic regression. Similar to the outcome of preterm delivery, both PCR and culture exhibited near-complete positive correlation: all but two PCR-positive cases, and all but one culture-positive case were associated with neonatal morbidity and mortality (Supporting Table S2). Although this led to wide confidence limits in the logistic regression models, the magnitude of the odds ratios suggests a significant association for both PCR and culture [adjusted OR 5.2 (95% CI, 0.84 to 32) and 7.1 (95% CI, 0.66 to 76), respectively] (Table 1).

### Temporal and dose response associations

To evaluate the association of microbial DNA with timing of delivery, results from end-point PCR and culture were correlated with the interval from amniocentesis to delivery. Kaplan-Meier survival estimates, censored for subjects in whom labor was augmented, and evaluated for differences by means of the Mantel-Haenszel log-rank test, revealed an overall association between a positive test result (PCR and/or culture) and a shortened amniocentesis-to-delivery interval (P<0.001) (Figure 4B). Pairwise comparison of the two cohorts who were positive by one method alone (i.e., P+/C− vs. P−/C+) revealed no difference in the amniocentesis-to-delivery interval of subjects who were positive only by PCR as compared with those who were positive only by culture (P = 0.5), indicating that a positive PCR alone is as significant as a positive culture alone (Figure 4B). However, pairwise comparison of the two cohorts testing positive by culture (i.e., P+/C+ vs. P−/C+) demonstrated a shorter amniocentesis-to-delivery interval for subjects who were positive by both culture and PCR as compared with those who were positive by culture but negative by PCR (P = 0.025, false discovery rate 26/1000) (Figure 4B). These unexpected findings support independent prognostic value for PCR even in the setting of a positive culture. The positive predictive value of PCR or culture for delivery within one day of amniocentesis was approximately 68% for either method alone, and 100% for both methods combined (Table 2). Cox proportional hazards regression, adjusted for nine covariates, estimated the hazard ratio of a positive PCR to be 4.6 (95% CI, 2.2 to 9.5), and of a positive culture to be 6.4 (95% CI, 2.8 to 15) (Supporting Table S3).

**Figure 5:**
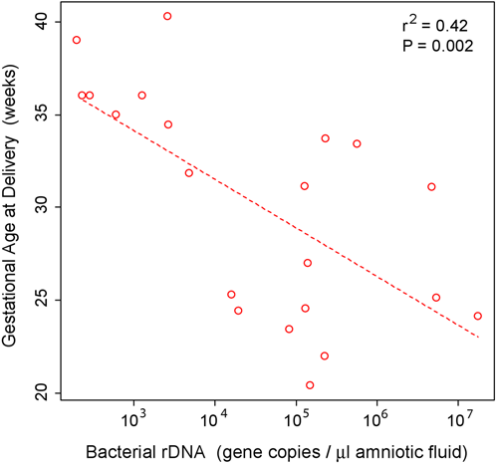
Gestational Age at Delivery as a Function of Bacterial rDNA Concentration. Data are for samples yielding detectable results within the sensitivity and dynamic range of the real-time PCR assay (∼250–1×10^8^ genes/µl amniotic fluid).

To evaluate dose-response associations, bacterial rDNA concentration was estimated by broad-range real-time PCR, and correlated with gestational age at delivery. Regression modeling revealed a significant correlation (r^2^ = 0.42; P<0.002) (Figure 5).

## Discussion

This study revealed significant associations between the presence and quantity of microbes or their DNA in amniotic fluid, and preterm delivery or other clinically-relevant outcomes. Microbial prevalence, as revealed by means of end-point PCR and culture combined (15%), was 56% higher than that found by culture alone (9.6%), which is the conventional diagnostic approach. PCR detected microbial DNA in culture-negative samples despite the limited sample volumes available for DNA extraction (200 µl per patient), and the fact that PCR was performed 2 to 6 years after sample collection and culturing, which may have limited the number of PCR-positive samples owing to DNA degradation [24]. The prevalence of a positive amniotic fluid culture/PCR was inversely related to gestational age at delivery, reflecting a previously-reported pattern [9]. Importantly, compared to both methods combined, culture exhibited a false negative rate of 27% (3/11) in subjects delivering before 25 weeks (Figure 4A), a cohort with a neonatal mortality rate exceeding 70% in meta-analyses [25]. This underscores the need for improved diagnostic assays in this setting.

The microbial diversity revealed by end-point PCR and culture combined (18 taxa) was almost twice as rich as that found by culture alone (11 taxa). Among the taxa identified by PCR, but not culture, were phylotypes that would not have been predicted from prior studies. One taxon appeared to be a novel species, and possibly a novel genus. The sequence of this taxon (clone PL036-b24; GenBank accession no. EU932745) clustered nearest the genus *Leptotrichia* within the phylum *Fusobacteria* (Figure 2). Two other fastidious members of this phylum went undetected by culture: *Leptotrichia amnionii* (n = 2) and *Sneathia* (formerly *Leptotrichia*) *sanguinegens* (n = 4). The latter was the third most frequently encountered taxon overall (Figure 2). Both taxa were provided valid species descriptions only recently [26], [27]. Sporadic reports of their detection in peripartum bacteremia [28]–[30], pyosalpinx [31], [32], and in amniotic fluid [27], [33], [34] associated with various clinical syndromes, suggest they possess pathogenic potential. *L. amnionii* has also been associated with bacterial vaginosis [14], a condition conferring an approximately two-fold risk of preterm delivery [35]–[37]. This study extends support for *Leptotrichia* sp., *Sneathia* sp. and related taxa as intra-amniotic pathogens, and suggests that their prevalence in the setting of preterm labor is underestimated. For other bacterial phylotypes detected only by PCR (*Delftia acidovorans* [Genbank accession no. EU932748], uncultured *Bacteroidetes* bacterium [Genbank accession no. EU932749], and *Neisseria cinerea* [Genbank accession no. EU932746]), we found no reports of their previous detection in either human amniotic fluid or maternal-fetal membranes.

A single fungal phylotype, *Candida albicans*, was detected in two subjects, both of whom were PCR-positive, and one of whom was culture-positive. We detected no members of *Archaea*, the most recently characterized domain of life [38]. This suggests that if archaea are capable of amniotic cavity invasion, this occurs rarely or involves abundance levels below the detection threshold of our assays.

Prior studies have applied species-specific [10], [11], [39]–[44] or broad-range [33], [34], [45]–[47] PCR to amniotic fluid, within various clinical settings. These early investigations yielded insights but had limitations. Indeed, the broad-range studies involved small sample sizes, and included rDNA sequencing that was limited [33], shallow (one sequence per positive PCR reaction) [34], [46], or absent [45], [47]. These studies also lacked measures of microbial abundance. Cumulatively, however, these important studies demonstrated the sensitivity of PCR in this setting, including the potential for detecting fastidious taxa such as *Ureaplasma* spp., and revealed associations with certain outcomes.

To overcome limitations of prior studies, we analyzed 166 subjects for microbes belonging to three broad taxonomic groups. Microbial diversity was assessed by sequencing up to 24 clones per subject. Microbial rDNA abundance was measured in parallel. Using this approach, we demonstrated greater microbial diversity than expected from prior studies. Compared to findings from culture, the molecular detection of these diverse microbes exhibited equivalent associations with indices of host inflammation, and with adverse gestational and neonatal outcomes.

Our study does not prove causation. To do so for multifactorial biological phenomena often entails successive investigations that expand knowledge within a coherent framework until sufficient evidence accumulates. Any single framework, however, may have limitations. The postulates of Robert Koch, for example, cannot be fulfilled for uncultivated pathogens [48]. However, an alternative causal framework based on epidemiologic guidelines proposed by Sir Austin Bradford Hill [49] may be particularly appropriate for molecular investigations [16]. Of Hill's nine criteria, our broad-range molecular findings demonstrate statistical support for three: strength of association, temporality and biological gradient. Additionally, our study augments a fourth general criterion of consistency of association (similar findings over multiple investigations using different methodology), and conforms to criteria of coherence, plausibility, analogy and experimentation as they pertain to intra-amniotic infection [8], [50].

Despite this study's progress in defining the microbial diversity associated with preterm delivery, much likely awaits discovery. Many detected phylotypes, such as the uncultivated *Bacteroidetes* bacterium (GenBank accession no. EU932749) and the previously-uncharacterized *Fusobacteria* bacterium (GenBank accession no. EU932745), were singletons that have been rarely, if ever, reported from human amniotic fluid. It can thus be inferred, based on ecological approaches for estimating species richness in sampled environments [51], that the amniotic cavity remains a largely unexplored niche.

This study has some limitations, some of which could affect results from molecular assays disproportionately, thus potentially contributing to a negative PCR in some culture-positive cases. First, DNA may have degraded during the interval from amniocentesis to molecular analysis (range, 2–6 years) [24], rendering it non-amplifiable in some samples. Second, the starting sample volume of 200 µl was probably suboptimal for use with broad-range PCR assays, which are less sensitive than species-specific PCR assays. Third, molecular findings may have been affected by PCR inhibition [52], or by biases in DNA extraction [52], PCR amplification efficiency [52], or PCR primer specificity [53]. Fourth, our approach may have failed to detect phylotypes present in polymicrobial samples at low relative abundance. Last, we did not target viruses or non-fungal eukaryotic microbes.

In conclusion, among patients with spontaneous preterm labor and intact membranes, the amniotic cavity harbors a greater diversity of microbes than previously suspected, including uncultivated, previously-uncharacterized taxa. The strength, temporality and gradient with which detected microbes were associated with clinically relevant outcomes, including preterm delivery, suggest a causal relationship. Despite these insights, the microbial census of the amniotic cavity is unfinished. Taken together, our findings support a contributory role for occult intra-amniotic infection in preterm delivery and its neonatal sequelae, and argue for further large-scale prospective molecular investigations.

## Supporting Information

Materials and Methods S1 Supporting Materials and Methods
(0.04 MB DOC)

Figure S1 Approach to Amniotic Fluid Analysis. Results reported in this study are shaded either grey (conventional analyses) or blue (molecular analyses). *PCR assays targeted the domain *Bacteria*, domain *Archaea* and the fungal division of *Eukarya*.
(0.28 MB TIF)

Table S1 Baseline Subject Characteristics According to Results of PCR and Culture of Amniotic Fluid.
(0.04 MB DOC)

Table S2 Characteristics of Individual Subjects Who Tested Positive by PCR or Culture.
(0.15 MB DOC)

Table S3 Association of Demographic and Microbiologic Variables with Shortened Amniocentesis-to-Delivery Interval.
(0.03 MB DOC)

Table S4 Broad-range PCR Assays Used in this Study.
(0.06 MB DOC)
