# Supplementary material for: Microbial Prevalence, Diversity and Abundance in Amniotic Fluid During Preterm Labor: A Molecular and Culture-Based Investigation
Source: PLoS One. 2008 Aug 26;3(8):e3056. doi: 10.1371/journal.pone.0003056 (PMC2516597; doi:10.1371/journal.pone.0003056)
Supplement: Materials and Methods S1 — Supporting Materials and Methods (0.04 MB DOC) [file pone.0003056.s001.doc]

Supporting Materials and Methods S1

**Culture of amniotic fluid**. Amniotic fluid was centrifuged for 10 minutes at 2000 RPM, the sediment resuspended in 1 cc of supernatant, and the solution plated onto chocolate agar, 5% sheep's blood agar, and MacConkey’s agar. Plates were incubated at 35ºC in aerobic and anaerobic conditions. To maximize recovery of genital mycoplasmas, amniotic fluid was also plated onto A7 differential agar and A7 diphasic enrichment tubes (Micro Diagnostics, Inc. Lombard, IL), and incubated anaerobically at 35ºC. All cultures were incubated for a minimum of 4 days. Isolated microbes were identified by MicroScan® (Dade Behring, Deerfield, IL) and, when appropriate, adjunctive biochemical and serotyping methods.

**Interleukin-6 (IL-6) immunoassays.** Specific and sensitive enzyme-linked immunoassays (R&D Systems, Minneapolis, MN) were used to determine concentrations of IL-6 in human amniotic fluid. These assays were specifically validated for use with human amniotic fluid in our laboratory prior to the conduction of this study. Validation included spike and recovery experiments, which produced parallel curves indicating that amniotic fluid constituents did not interfere with antigen-antibody binding. Briefly, amniotic fluid samples were incubated in duplicate wells of microtiter plates that were pre-coated with IL-6 specific antibodies. During this incubation IL-6 present in the standards or amniotic fluid samples were immobilized by their specific pre-coated antibodies (form antigen antibody complexes). Repeated washing and aspiration removed all other unbound materials from the assay plate. This step was followed by incubation with a specific antibody-enzyme reagent. Following a wash to remove excess and unbound materials, a substrate solution was added to the wells of the microtiter plate and color developed in proportion to the amount of IL-6 bound in the initial step of the individual assays. The color development was stopped with the addition of an acid solution, and color intensity was read using a programmable microtiter plate spectrophotometer (SpectraMax M2, Molecular Devices, Sunnyvale, CA). The concentrations of IL-6 in amniotic fluid samples were determined by interpolation from individual standard curves composed of human IL-6. The calculated inter-assay and intra-assay coefficients of variation (CV) for IL-6 in our laboratory were 4.2% and 3.7% respectively. The detection limit (sensitivity) was calculated to be 1.67 pg/ml.

**Genomic DNA extraction from amniotic fluid.** Amniotic fluid not required for clinical purposes was centrifuged at 700 x g for 10 minutes at 4°C, aliquoted into gamma-irradiated nonpyrogenic DNase/RNase-free cryovials (Corning, Acton, MA), and immediately frozen at -70ºC. Frozen samples were shipped on dry ice to Stanford, CA, where they were stored at -70ºC until DNA extraction. The interval from amniocentesis to DNA extraction ranged from ~2-6 years. After thawing, 200 µl of each amniotic fluid sample was centrifuged for 15 min at 14,000 x g, the supernatant decanted and the pellet resuspended in 180 µl of lysozyme solution (20mg/ml) (Sigma-Aldrich, St. Louis, MO). After incubation at 37ºC for 30 min, total genomic DNA was extracted from the resuspension by means of the QIAamp® DNA Mini Kit (Qiagen, Foster City, CA) according to the manufacturer’s protocol including the optional addition of 4 µl of RNase A (100 mg/ml), but modified to extend the 70ºC incubation in QIAamp® AL buffer from 10 to 30 min. Extracted DNA was eluted in a final volume of 100 µl of QIAamp® AE buffer and stored at -20ºC or colder until thawing for molecular analyses. Mock extraction blanks were systematically incorporated to monitor potential contamination (see “Contamination prevention protocol”).

**Qualitative analysis by broad-range end-point PCR.** Extracted DNA from each amniotic fluid sample was analyzed by means of three separate end-point PCR assays, each of which was designed to specifically amplify ribosomal DNA (rDNA) of bacteria, fungi and archaea, respectively (Supporting Table S4). For all assays, each 50-µl reaction consisted of 1× PCR buffer II (Applied Biosystems, Foster City, CA), 1.5 mM MgCl_2_, 0.05% Triton X-100, 20 mM tetramethylammonium chloride, 0.1 mM concentrations of each deoxyribonucleoside triphosphate, 0.4 µM concentrations of each commercially-synthesized primer (Integrated DNA Technologies, Coralville, IA), 1 U of AmpliTaq Gold® DNA polymerase (Applied Biosystems), and 5 µl of prepared DNA template. PCR reactions were carried out in a GeneAmp® 9700 PCR thermocycler (Applied Biosystems) using conditions of 95°C for 10 min, followed by 35 cycles of 95°C for 30 s, 55°C for 30 s, and 72°C for 30 s (45 s in the fungal assay), followed by a final step at 72°C for 8 min. A positive control plasmid containing rDNA from the appropriate microbial group (i.e., bacteria, fungi, or archaea) was included at known ten-fold serial dilutions on each 96-well PCR reaction plate to measure the sensitivity of each experiment. Approximate assay sensitivity was (molecules / µl prepared DNA template): bacterial - 10^3^; fungal - 10^3^; archaeal - 10^4^.

From each PCR reaction well, 15 µl of sample was electophoresed through a 1.5% (wt/vol) Tris-acetate-EDTA (TAE)-agarose gel containing ethidium bromide (0.33 mg/l). Amplicons producing visible bands upon UV transillumination were excised and purified with the QIAquick® gel extraction kit (Qiagen) according to the manufacturer’s protocol. Purified amplicons were ligated into the pCR4.0 vector, and transformed into *E. coli* TOP10 cells by using the TOPO-TA cloning kit (Invitrogen, Carlsbad, CA) according to the manufacturer’s instructions. After overnight incubation at 37ºC on selective media, up to 24 clones per positive PCR reaction were screened for appropriate insert size. Inserts from positive recombinants were sequenced on an AB 3730xl sequencer (Applied Biosystems) using BigDye® Terminator reagents (Applied Biosystems).

**Sequence alignment and phylogenetic analysis.** Forward and reverse sequences were assembled into contigs using the SeqMan program of the DNAstar software suite (Madison, WI; http://www.dnastar.com/). Contigs assembled from prokaryotic sequences were imported into the Arb software package [8] and aligned against small subunit rRNA sequences from the Ribosomal Database Project (RDP) II, release 9.1 (<http://rdp.cme.msu.edu/index.jsp>). Alignments were manually inspected and edited based on the original chromatograms and inserted into the RDP phylogeny according to a maximum parsimony algorithm. Sequences with no close relative in the RDP database were queried against NCBI’s GenBank database using a basic local alignment search tool (BLAST) algorithm^9^ to determine approximate phylogenetic affiliation, and their closest neighbors were added to the alignment. Sequences without close neighbors were screened using RDP’s ‘Chimera Check’ program (<http://35.8.164.52/cgis/chimera.cgi?su=SSU>). After removal of chimeric, vector, human, and poor-quality sequences from the alignment, a maximum-likelihood tree was generated based on Olsen correction and 621 non-ambiguous filter positions. Phylotypes were defined by using a 99% cut off criterion. Contigs assembled from fungal sequences were queried against NCBI’s GenBank database using a BLAST algorithm [9] and assigned a taxonomic designation to the top hit if the percent similarity was >99% and the expected (E) value was 0.0.

**Quantitative analysis by broad-range real-time PCR**. Extracted DNA from each sample was analyzed by means of three separate real-time PCR assays, each of which was designed to specifically amplify and quantify ribosomal DNA (rDNA) of the same groups targeted by end-point PCR (Supporting Table S4). Each 20-µl reaction consisted of 1x TaqMan® Universal PCR Master Mix with AmpErase® UNG (Applied Biosystems), 1 U of AmpliTaq Gold® DNA polymerase (Applied Biosystems), 0.9 µM of each commercially-synthesized primer (Integrated DNA Technologies), 0.2 µM of TaqMan® probe, and 2 µl of prepared DNA template. Amplifications were carried out in a Prism 7900HT Sequence Detection System (SDS) (Applied Biosystems), using the following thermal cycling protocol: 95°C for 10 min, followed by 40 cycles of 95°C for 30 s, 55°C for 30 s, 60°C for 45 s, 65°C for 15 s, and 72°C for 15 s. A positive control plasmid containing rDNA from the appropriate microbial group (i.e., bacteria, fungi, or archaea) was included in triplicate at known ten-fold serial dilutions on each 384-well reaction plate to generate standard curves. Absolute rDNA abundance was estimated from the standard curves using SDS software version 2.1 (Applied Biosystems) with the baseline and cycle threshold set according to the manufacturer’s recommendations. The sensitivity and dynamic range of each assay was approximately 250 - 10^8^ rDNA molecules / µl prepared DNA template.

**Contamination prevention protocol**. Strategies to prevent, detect and neutralize potential contamination were systematically implemented at critical steps [10]. To screen for background DNA in essential supplies, the reagents and buffers used for DNA extractions or PCR reactions were assayed by means of bacterial and fungal real-time PCR. To avoid cross contamination during sample processing and experimental setup, a “white-grey-black” workflow scheme utilizing three separate rooms was implemented. PCR reactions (minus template DNA) were set up in the “white” room, genomic DNA extractions as well as the addition of template DNA to PCR reactions occurred in the “grey” room, and PCR reactions were carried out in the “black” room; flow of materials from “darker” to “lighter” rooms was prohibited. To monitor for contamination that may have circumvented this proactive approach, a DNA-extraction “mock” was generated for each batch of ~18 processed samples (total of 11 mocks for 203 samples). Mocks consisted of ultrapure sterile water that was processed in an identical manner as study samples. Each time a given sample was analyzed by a downstream molecular assay, its corresponding mock was also assayed in the same experiment. Every PCR experiment also included a minimum of one reagent negative control and one aliquot negative control. No extraction mock or negative control yielded a visible band upon gel electrophoresis of its end-point PCR reaction products. Finally, a dUTP/uracil N-glycosylase strategy was incorporated into real-time PCR reactions to prevent reamplification of carryover amplicons.

**Statistical analysis.** Additional details of statistical analyses are as follows. For multiple regression, final model selection was informed by the results of univariate analysis, as well as stepwise analysis based on both forward addition and backward elimination of covariates. For multiple pairwise comparisons, significant P values were evaluated by bootstrap resampling of 1000 replicates to calculate a false discovery rate representing the likelihood that the applicable P value occurred by chance (i.e., Type I error). Because the maximum number of pairwise comparisons in any analysis was three, bootstrapping was applied only to comparisons that had a P value between 0.001 and 0.05.

**References**

1. Palmer C, Bik EM, DiGiulio DB, Relman DA, Brown PO (2007) Development of the Human Infant Intestinal Microbiota. PLoS Biol 5: e177.

2. Wilson KH, Blitchington RB, Greene RC (1990) Amplification of bacterial 16S ribosomal DNA with polymerase chain reaction. J Clin Microbiol 28: 1942-1946.

3. Gardes M, Bruns TD (1993) ITS primers with enhanced specificity for basidiomycetes--application to the identification of mycorrhizae and rusts. Mol Ecol 2: 113-118.

4. White TJ, Burns T, Lee S, Taylor J (1990) Amplification and sequencing of fungal ribosomal RNA genes for phylogenetics. In: Innis MA, Gelfand DH, Sninsky JJ, White TJ, editors. PCR Protocols A guide to methods and applications. San Diego, CA: Academic Press, Inc. pp. 315-322.

5. Lepp PW, Brinig MM, Ouverney CC, Palm K, Armitage GC, et al. (2004) Methanogenic Archaea and human periodontal disease. Proc Natl Acad Sci U S A 101: 6176-6181.

6. DeLong EF (1992) Archaea in coastal marine environments. Proc Natl Acad Sci U S A 89: 5685-5689.

7. Brinig MM, Lepp PW, Ouverney CC, Armitage GC, Relman DA (2003) Prevalence of bacteria of division TM7 in human subgingival plaque and their association with disease. Appl Environ Microbiol 69: 1687-1694.

8. Ludwig W, Strunk O, Westram R, Richter L, Meier H, et al. (2004) ARB: a software environment for sequence data. Nucleic Acids Res 32: 1363-1371.

9. Altschul SF, Gish W, Miller W, Myers EW, Lipman DJ (1990) Basic local alignment search tool. J Mol Biol 215: 403-410.

10. Borst A, Box AT, Fluit AC (2004) False-positive results and contamination in nucleic acid amplification assays: suggestions for a prevent and destroy strategy. Eur J Clin Microbiol Infect Dis 23: 289-299.
