# Supplementary figures and images for: Microbial Prevalence, Diversity and Abundance in Amniotic Fluid During Preterm Labor: A Molecular and Culture-Based Investigation
Source: PLoS One. 2008 Aug 26;3(8):e3056. doi: 10.1371/journal.pone.0003056 (PMC2516597; doi:10.1371/journal.pone.0003056)

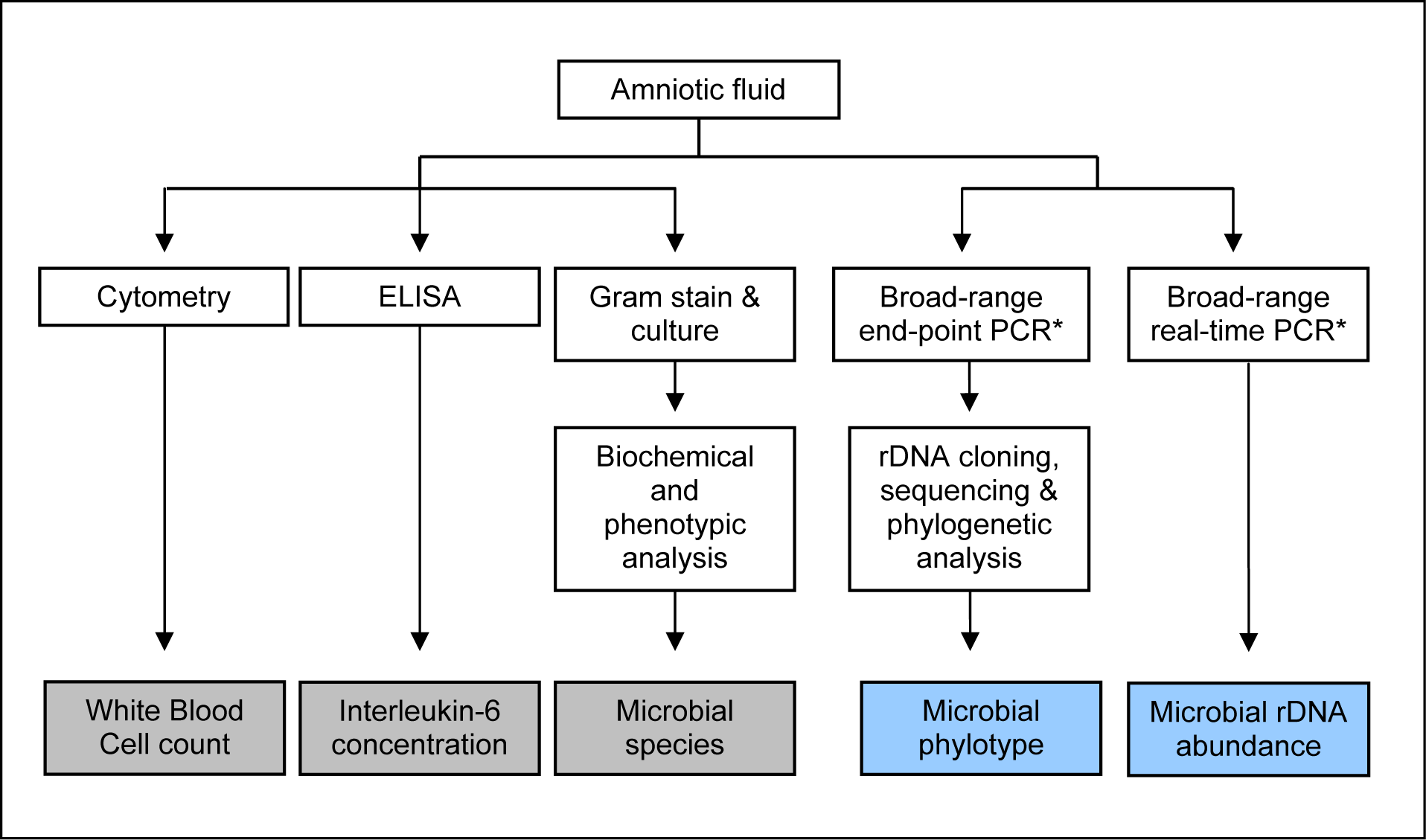

Supplement: Figure S1 — Approach to Amniotic Fluid Analysis. Results reported in this study are shaded either grey (conventional analyses) or blue (molecular analyses). *PCR assays targeted the domain Bacteria, domain Archaea and the fungal division of Eukarya. (0.28 MB TIF) [file pone.0003056.s002.tif]
