# Supplementary material for: Microbial Prevalence, Diversity and Abundance in Amniotic Fluid During Preterm Labor: A Molecular and Culture-Based Investigation
Source: PLoS One. 2008 Aug 26;3(8):e3056. doi: 10.1371/journal.pone.0003056 (PMC2516597; doi:10.1371/journal.pone.0003056)
Supplement: Table S1 — Baseline Subject Characteristics According to Results of PCR and Culture of Amniotic Fluid. (0.04 MB DOC) [file pone.0003056.s003.doc]

|  | **Culture (-)** | **Culture (+)** | **Culture (-)** | **Culture (+)** | **P-value*** |
| --- | --- | --- | --- | --- | --- |
|  | **PCR (-)** | **PCR (-)** | **PCR (+)** | **PCR (+)** |  |
| **Characteristic** | n=141 | n=6 | n=9 | n=10 |  |
|  |  |  |  |  |  |
| Maternal age (yrs, mean ±SD) | 23.6 ± 6.0 | 23.2 ± 2.7 | 25.2 ± 5.6 | 24.8 ± 5.4 | P=0.61 |
|  |  |  |  |  |  |
| Gestational age at amniocentesis | 29.7 (18.6-34.6) | 24.4 (20.3-25.9) | 27 (23.9-32.9) | 24.6 (20.3-33.7) | P=0.02 |
| (wks, median and range) |  |  |  |  |  |
|  |  |  |  |  |  |
| Cervical dilatation at | 2.2 ± 1.5 | 3.1 ± 1.0 | 3.9 ± 1.8 | 2.9 ± 2.0 | P=0.03 |
| amniocentesis (cm, mean ±SD) |  |  |  |  |  |
|  |  |  |  |  |  |
| Nulliparous (number, percent) | 35 (25%) | 2 (33%) | 0 | 2 (20%) |  |
|  |  |  |  |  |  |

*P values calculated by Kruskal-Wallis ANOVA
