# Supplementary material for: Microbial Prevalence, Diversity and Abundance in Amniotic Fluid During Preterm Labor: A Molecular and Culture-Based Investigation
Source: PLoS One. 2008 Aug 26;3(8):e3056. doi: 10.1371/journal.pone.0003056 (PMC2516597; doi:10.1371/journal.pone.0003056)
Supplement: Table S2 — Characteristics of Individual Subjects Who Tested Positive by PCR or Culture. (0.15 MB DOC) [file pone.0003056.s004.doc]

| **Case** | **Gest- ational age at delivery (weeks)** | **Amnio- centesis to delivery interval (days)** | **Birth weight (g)** | **Amniotic Fluid WBCs (cells/ mm3)** | **Amniotic Fluid IL-6 (ng/ml)** | **Histologic chorio- amnionitis*** | **Funisitis†** | **Neonatal Morbidity‡** | **Culture** | **PCR** | **GenBank accession number(s) of ribosomal DNA sequences§** |
| --- | --- | --- | --- | --- | --- | --- | --- | --- | --- | --- | --- |
| 1 | 31.1 | 7 | 1670 | 50 | 11 | Present | Present | Y | (negative) | *Ureaplasma* sp. | EU932691 - EU932695 |
| 2 | 25.3 | 1 | 520 | 1120 | 155 | Present | Present | Y | (negative) | *Ureaplasma* sp. | EU932696 - EU932703 |
| 3 | 33.4 | 1 | 2300 | 720 | 57 | Present | Present | Y | *Mycoplasma hominis* | *Mycoplasma hominis* | EU932704 - EU932712 |
| 4 | 27.0 | 0 | 824 | 2100 | 297 | Present | Present | Y | *Fusobacterium nucleatum* | *Fusobacterium nucleatum* | EU932713 - EU932720 |
|  |  |  |  |  |  |  |  |  |  | *Sneathia sanguinegens* | EU932721 - EU932722 |
| 5 | 34.0 | 8 | 1940 | 190 | 64 | NA | NA | Y | (negative) | *Candida albicans* | EU932723 - EU932730 |
| 6 | 20.4 | 1 | 280 | 7 | 1101 | Present | Present | NA (pregnancy loss) | *Fusobacterium nucleatum* | *Fusobacterium nucleatum* | EU932731 - EU932744 |
|  |  |  |  |  |  |  |  |  |  |  |  |
| 7 | 31.1 | 0 | 1530 | 20 | 2 | Absent | Absent | Y | (negative) | uncultivated *Fusobacteria* |  |
|  |  |  |  |  |  |  |  |  |  | bacterium [clone PL036-b24] | EU932745 |
|  |  |  |  |  |  |  |  |  |  | *Neisseria cinerea* | EU932746 |
|  |  |  |  |  |  |  |  |  |  | *Streptococcus mitis* | EU932747 |
|  |  |  |  |  |  |  |  |  |  | *Defltia acidovorans* | EU932748 |
|  |  |  |  |  |  |  |  |  |  | uncultivated *Bacteroidetes* |  |
|  |  |  |  |  |  |  |  |  |  | bacterium | EU932749 |
| 8 | 27.3 | 2 | 900 | 6050 | 296 | Present | Present | Y | (negative) | *Streptococcus mitis* | EU932750 - EU932756 |
| 9 | 24.1 | 0 | 640 | 1800 | 66 | Present | Present | Y | *Streptococcus agalactiae* | *Streptococcus agalactiae* | EU932757 - EU932765 |
| 10 | 25.9 | 6 | 1060 | 168 | 82 | Absent | Absent | Y | *Bacillus* species, not | (negative) | NA |
|  |  |  |  |  |  |  |  |  | anthracis |  |  |
| 11 | 21.0 | 5 | 400 | 120 | 11 | Present | Present | Y | *Peptostreptococcus* | (negative) | NA |
|  |  |  |  |  |  |  |  |  | *asaccharolyticus* |  |  |
| 12 | 24.4 | 4 | 800 | 520 | 316 | Present | Present | Y | (negative) | *Ureaplasma* sp. | EU932766 - EU932771 |
| 13 | 25.1 | 3 | 1040 | 72 | 46 | Present | Present | Y | *Peptostreptococcus* sp. | (negative) | NA |
| 14 | 33.7 | 0 | 1760 | 515 | 120 | Present | Present | Y | *Gardnerella vaginalis* | *Sneathia sanguinegens* | EU932772 - EU932780 |
| 15 | 24.4 | 4 | 600 | 92 | 28 | Present | Present | Y | (negative) | *Sneathia sanguinegens* | EU932781 - EU932785 |
|  |  |  |  |  |  |  |  |  |  | *Leptotrichia amnionii* | EU932786 |
|  |  |  |  |  |  |  |  |  |  | *Prevotella sp.* | EU932787 |
| 16 | 25.1 | 0 | 709 | 5500 | 170 | Present | Present | Y | *Prevotella melaninogenica* | *Prevotella* sp. | EU932788 - EU932789 |
|  |  |  |  |  |  |  |  |  | *Ureaplasma urealyticum* | *Sneathia sanguinegens* | EU932790 - EU932796 |
|  |  |  |  |  |  |  |  |  | *Gardnerella vaginalis* | *Leptotrichia amnionii* | EU932797 |
| 17 | 31.1 | 10 | 1550 | 12 | 232 | Present | Present | Y | (negative) | *Streptococcus mitis* | EU932798 - EU932810 |
| 18 | 21.1 | 1 | 400 | 2 | 17 | Present | Present | NA (pregnancy loss) | *Ureaplasma urealyticum* | (negative) | NA |
| 19 | 24.6 | 0 | 600 | 40 | 166 | Present | Present | Y | (negative) | *Fusobacterium nucleatum* | EU932811 - EU932818 |
| 20 | 24.1 | 0 | 650 | 26 | 150 | Present | Present | Y | *Fusobacterium nucleatum* | (negative) | NA |
|  |  |  |  |  |  |  |  |  | *Ureaplasma urealyticum* |  |  |
| 21 | 23.4 | 1 | 560 | 260 | 461 | Present | Absent | Y | *Fusobacterium nucleatum* | *Fusobacterium nucleatum* | EU932819 - EU932835 |
| 22 | 28.4 | 18 | 1140 | 0 | 20 | Present | Present | Y | *Staphylococcus* sp., | (negative) | NA |
|  |  |  |  |  |  |  |  |  | coagulase-negative |  |  |
| 23 | 22.1 | 1 | 525 | 340 | 328 | Present | Present | Y | *Candida albicans* | *Candida albicans* | EU932836 - EU932843 |
| 24 | 31.9 | 0 | 1660 | 10 | 238 | Present | Present | Y | *Lactobacillus* species | *Lactobacillus* sp. | EU932844 - EU932847 |
| 25 | 22.0 | 1 | 420 | 153 | 368 | Present | Present | Y | *Fusobacterium nucleatum* | *Fusobacterium nucleatum* | EU932848 - EU932861 |

WBCs denotes white blood cells

IL-6 denotes interleukin-6

NA - not applicable

* Histologic chorioamnionitis was diagnosed based on the presence of inflammatory cells in the chorionic plate and/or chorioamniotic membranes.

† Funisitis was defined as the presence of neutrophils in the wall of the umbilical vessels and/or Wharton’s jelly.

‡ Neonatal morbidity and mortality was defined as the presence of any one or more of the following: bronchopulmonary dysplasia, respiratory distress syndrome, necrotizing enterocolitis, intraventricular hemorrhage of grade ≥3, sepsis, respiratory failure requiring mechanical ventilation or neonatal death.

§ One rDNA consensus sequence was submitted to GenBank for each phylotype detected by PCR in each subject. In addition, when rDNA sequence microheterogeneity was present among two or more clones of the same phylotype, a single representative of each unique rDNA sequence also was submitted to GenBank. To prevent spurious submissions resulting from DNA sequencing errors, sequences that varied from the consensus were considered unique only when a given nucleotide polymorphism was supported by both the forward and reverse reads, with verification by manual inspection of the corresponding electropherograms.
