# Supplementary material for: Microbial Prevalence, Diversity and Abundance in Amniotic Fluid During Preterm Labor: A Molecular and Culture-Based Investigation
Source: PLoS One. 2008 Aug 26;3(8):e3056. doi: 10.1371/journal.pone.0003056 (PMC2516597; doi:10.1371/journal.pone.0003056)
Supplement: Table S3 — Association of Demographic and Microbiologic Variables with Shortened Amniocentesis-to-Delivery Interval. (0.03 MB DOC) [file pone.0003056.s005.doc]

| **Variable** | **Adjusted Hazard Ratio** |
| --- | --- |
|  | ***(95% confidence interval)*** |
|  |  |
| Positive PCR of amniotic fluid | 4.6 (2.2-9.5) |
|  |  |
| Positive culture of amniotic fluid | 6.4 (2.8-15) |
|  |  |
| Gestational age at amniocentesis | 1.1 (1.1-1.2) |
|  |  |
| Cervical Dilatation | 1.5 (1.3-1.7) |
|  |  |
| Maternal age | 1.0 (1.0-1.1) |
|  |  |
| African-American race | 0.7 (0.4-1.2) |
|  |  |
| Cigarette smoking | 0.8 (0.5-1.4) |
|  |  |
| Drug use | 1.9 (1.0-3.6) |
|  |  |
| Nulliparity | 1.4 (0.9-2.2) |
|  |  |

*Estimated by means of Cox proportional hazards modeling after adjusting for the covariates shown.
