# Supplementary material for: Microbial Prevalence, Diversity and Abundance in Amniotic Fluid During Preterm Labor: A Molecular and Culture-Based Investigation
Source: PLoS One. 2008 Aug 26;3(8):e3056. doi: 10.1371/journal.pone.0003056 (PMC2516597; doi:10.1371/journal.pone.0003056)
Supplement: Table S4 — Broad-range PCR Assays Used in this Study. (0.06 MB DOC) [file pone.0003056.s006.doc]

| **Endpoint PCR** | **Oligonucleotide** | **Purpose** | **Sequence (5' -> 3')** | **Gene** | **Reference** |
| --- | --- | --- | --- | --- | --- |
| **assay specificity** | **name** |  |  | **target** |  |
|  |  |  |  |  |  |
| Bacteria | Bact-8FM | Forward primer | AGAGTTTGATCMTGGCTCAG | 16S rDNA | [1] |
|  | Bact-806R | Reverse primer | GGACTACCAGGGTATCTAAT | 16S rDNA | [2] |
|  |  |  |  |  |  |
| Fungi | ITS1F-F | Forward primer | CTTGGTCATTTAGAGGAAGTAA | 18S rDNA | [3] |
|  | ITS4-R | Reverse primer | TCCTCCGCTTATTGATATGC | 28S rDNA | [4] |
|  |  |  |  |  |  |
| Archaea | Arch333F | Forward primer | TCCAGGCCCTACGGG | 16S rDNA | [5] |
|  | Arch958R | Reverse primer | YCCGGCGTTGAMTCCAATT | 16S rDNA | [6] |
|  |  |  |  |  |  |
| **Real-time PCR** |  |  |  |  |  |
| **assay specificity** |  |  |  |  |  |
|  |  |  |  |  |  |
| Bacteria | Bact-8FM | Forward primer | AGAGTTTGATCMTGGCTCAG | 16S rDNA | [1] |
|  | Bact-338K | TaqMan® probe* | CCAKACTCCTACGGGAGGCAGCAG | 16S rDNA | [1] |
|  | Bact-515R | Reverse primer | TTACCGCGGCKGCTGGCAC | 16S rDNA | [7] |
|  |  |  |  |  |  |
| Fungi | ITS1F-F | Forward primer | CTTGGTCATTTAGAGGAAGTAA | 18S rDNA | [3] |
|  | Probe5.8S | TaqMan® probe* | CATTTCGCTGCGTTCTTCATCGATG | 5.8S rDNA | modified from [4] |
|  | ITS4-R | Reverse primer | TCCTCCGCTTATTGATATGC | 28S rDNA | [4] |
|  |  |  |  |  |  |
| Archaea | Arch333F | Forward primer | TCCAGGCCCTACGGG | 16S rDNA | [5] |
|  | 515F | TaqMan® probe* | GTGCCAGCMGCCGCGGTAA | 16S rDNA | [7] |
|  | Arch958R | Reverse primer | YCCGGCGTTGAMTCCAATT | 16S rDNA | [6] |
|  |  |  |  |  |  |

* Conjugated on the 5' end to 6-carboxyfluorescein, and on the 3' end to 6-carboxy-tetramethylrhodamine
